# Perioperative Cardiac Complications and Evidence-Based Strategies for Their Management

**DOI:** 10.7759/cureus.95276

**Published:** 2025-10-23

**Authors:** Zubair Farooq, Saima Malik, Muttaib Bhat, Salik Farooq

**Affiliations:** 1 Cardiology, S.K.R Hospital and Trauma Center Private Limited, Pathankot, IND; 2 General Surgery, Government Medical College Hospital-Jammu, Jammu, IND; 3 General Surgery, Tairunnessa Memorial Medical College and Hospital, Dhaka, BGD; 4 General Medicine, Sher-i-Kashmir Institute of Medical Sciences, Srinagar, IND

**Keywords:** biomarkers, cardiovascular management, noncardiac surgery, perioperative cardiac complications, risk stratification

## Abstract

Perioperative cardiac complications remain a major cause of morbidity and mortality after noncardiac surgery. Their significance is growing as surgical populations age with increasing comorbidities. Patients with underlying cardiovascular disease (e.g., coronary artery disease, heart failure, arrhythmias) or other high-risk conditions (advanced age, diabetes, renal or cerebrovascular disease) face higher perioperative cardiac risk. Surgery-related factors (emergency or major vascular operations) and intraoperative hemodynamic perturbations (hypotension, tachycardia, bleeding) further amplify this risk. The pathophysiology involves a perioperative stress response - sympathetic activation, inflammation, hypercoagulability, and oxygen supply-demand imbalance - that can precipitate myocardial ischemia or injury (via either plaque rupture and thrombosis or demand mismatch), as well as arrhythmias or heart failure. Effective care relies on early risk stratification and tailored management. Clinical assessment and functional evaluation, aided by validated risk scores and biomarkers (natriuretic peptides, troponin), help identify high-risk patients. Preoperative optimization of cardiac conditions (angina or heart failure control) and continuation of cardioprotective therapies (statins, beta-blockers, antiplatelets when indicated) are important. Intraoperative strategies include vigilant monitoring and maintenance of hemodynamic stability, while postoperative surveillance (with telemetry and biomarker checks) allows prompt detection of ischemia or arrhythmias. Timely treatment of any cardiac event, together with an integrated multidisciplinary approach, is essential to mitigate complications and improve outcomes.

## Introduction and background

Cardiac complications in the perioperative period remain a major concern in non-cardiac surgery due to their frequency and severity [1]. Globally, more than 300 million major surgical procedures are performed each year, with perioperative mortality within 30 days estimated at around 1-2 % in adults over 45 years. Roughly half of these deaths are cardiovascular in origin, accounting for a substantial portion of postoperative morbidity and healthcare expenditure [2]. Reported incidence rates of perioperative cardiac events (e.g., myocardial infarction, heart failure, serious arrhythmias, or cardiac arrest) range from approximately 1% in low-risk surgical populations to as high as 5%-7% in higher-risk groups [2]. These cardiovascular complications are among the leading causes of postoperative morbidity and mortality, underscoring their clinical and public health significance. Notably, certain surgeries carry particularly elevated risk - for example, emergency procedures have roughly double the incidence of cardiac events compared to elective operations (on the order of ~5% vs ~3%) [2], and major orthopedic and general surgical procedures rank among those with the highest rates of perioperative cardiovascular complications [3].

Patients who experience a perioperative cardiac complication suffer substantial adverse outcomes. A postoperative myocardial infarction or acute cardiac event markedly increases short-term and long-term mortality risk and is associated with prolonged hospital stay and higher healthcare utilization [4]. In one large cohort study, surgical patients who sustained a perioperative organ injury (e.g., myocardial infarction) had an in-hospital mortality of 17%, compared to only 0.7% in those without such complications [4]. Equally concerning, many perioperative myocardial infarctions go unrecognized at the bedside - anesthesia and analgesia often blunt ischemic symptoms, and continuous ECG monitoring is not routinely employed, meaning transient ischemic changes are frequently missed [1]. Indeed, the advent of routine biomarker surveillance has revealed that a majority of perioperative myocardial injuries are clinically “silent.” The concept of myocardial injury after non-cardiac surgery (MINS), defined as an elevation in cardiac troponin following non-cardiac surgery in the absence of overt ischemic symptoms, has emerged to describe isolated troponin elevations indicative of cardiac injury without overt symptoms. Studies using postoperative troponin measurements have shown that the incidence of MINS is about 8%-19% with conventional troponin assays, and up to 20%-30% when high-sensitivity troponin assays are used. These occult events carry significant prognostic weight: even asymptomatic elevations in postoperative troponin are associated with a markedly higher 30-day mortality rate [1]. In other words, perioperative myocardial infarction - whether clinically apparent or detected only by biomarkers - portends a substantial increase in early postoperative morbidity and mortality, emphasizing the need for vigilant detection and management.

Given this reality, thorough preoperative risk stratification and early identification of at-risk patients are paramount. A number of patient-specific factors (advanced age, prior cardiovascular disease, poor functional capacity, diabetes, hypertension, etc.) and surgery-specific factors (high-risk surgeries, especially emergency or urgent cases) are known to predispose individuals to perioperative cardiac complications. For instance, advanced age and emergent surgery status each independently confer significantly elevated risk of cardiac events [2]. To quantify risk, clinicians rely on validated indices and algorithms. The Revised Cardiac Risk Index (RCRI), for example, is a widely used tool that estimates the 30-day risk of major cardiac complications (e.g., myocardial infarction or cardiac arrest) based on six clinical criteria (including history of ischemic heart disease, heart failure, cerebrovascular disease, diabetes, renal dysfunction, and high-risk surgery) [3]. Patients with multiple RCRI predictors face a sharply higher likelihood of perioperative major adverse cardiac events (MACE) - a composite outcome that includes myocardial infarction, cardiac arrest, or cardiac death compared to those with none. Landmark studies, such as the VISION, POISE, and METS trials, have further characterized perioperative cardiac risk, clarifying the prognostic role of biomarkers, perioperative ischemia, and functional capacity assessment in non-cardiac surgery [5]. Importantly, risk stratification is not based on clinical history alone - current guidelines emphasize a stepwise approach that incorporates assessment of functional capacity and judicious use of additional testing when indicated [5]. Both the American College of Cardiology/American Heart Association (ACC/AHA) and European Society of Cardiology (ESC) guidelines recommend reserving preoperative cardiac investigations (e.g., stress tests or imaging) for situations in which the results would meaningfully change management while proceeding with optimized medical therapy and appropriate monitoring [5]. Identifying high-risk patients preoperatively allows the perioperative team to optimize medical therapy (e.g., treating uncontrolled blood pressure or ischemia), consider cardiology consultation, and plan intraoperative anesthetic and hemodynamic strategies to mitigate cardiac stress.

Recent guidelines and innovations have also introduced new tools for risk assessment and early detection. The latest 2024 ACC/AHA perioperative cardiovascular guidelines, for instance, not only reinforce the traditional algorithm of clinical risk evaluation but also introduce the option of biomarker-based risk stratification in selected patients [5]. Measuring cardiac biomarkers, such as B-type natriuretic peptide (BNP or NT-proBNP) and high-sensitivity troponin before surgery, can aid in unmasking occult cardiac risk in patients with poor exercise tolerance or multiple risk factors [5]. In a large multicenter cohort, elevated preoperative NT-proBNP and high-sensitivity troponin T independently predicted postoperative MACE, supporting their integration into contemporary risk models [6]. An elevated preoperative BNP or troponin has been shown to predict a higher likelihood of postoperative cardiac events, and integrating these biomarkers into risk models is an area of active research and guideline discussion [6]. Similarly, there is growing interest in enhanced postoperative monitoring to detect complications as early as possible. Recent prospective data also show that routine postoperative troponin surveillance identifies otherwise silent myocardial injury in nearly one-fifth of high-risk surgical patients and is associated with improved early risk stratification and outcomes [7]. Some experts and recent guidelines have advocated for routine postoperative troponin surveillance in high-risk patients, given that timely detection of asymptomatic myocardial injury could prompt interventions (e.g., intensive monitoring, cardiology evaluation, or medical therapy optimization) to improve outcomes [5]. Parallel to these advances, novel risk prediction models have been developed to augment or even surpass older indices such as the RCRI. For example, the AUB-HAS2 score - which incorporates factors such as age ≥75, anemia (low hemoglobin), urgency of surgery, and known heart disease - has shown promise in outperforming RCRI for predicting cardiac complications in recent studies [3]. The continuous refinement of risk scores and the adoption of high-sensitivity diagnostic tools exemplify a broader push toward early, proactive identification of perioperative cardiac events.

Despite these efforts, perioperative cardiac complications remain a significant source of perioperative morbidity and mortality, particularly as the surgical population ages and more complex procedures are performed. Major orthopedic surgeries (e.g., hip fracture repairs or joint replacements) and extensive general surgical procedures often involve older patients with multiple comorbidities, placing them at especially high risk for cardiac events [3]. There is a clear need for heightened awareness, improved risk stratification, and evidence-based management strategies to address this ongoing challenge. In light of the incidence and impact of perioperative cardiac complications - and the evolving landscape of guidelines and preventive strategies - we present this comprehensive review. Here, we will examine the current evidence on the burden of perioperative cardiac complications in general and orthopedic surgery, discuss established and emerging methods for risk assessment (including clinical risk scores and biomarkers), and highlight recent guideline recommendations and innovations in monitoring and management. This review aims to summarize current evidence on the epidemiology, risk evaluation, and management of perioperative cardiac complications, emphasizing evidence-based preventive and monitoring strategies to improve surgical outcomes.

## Review

Perioperative cardiac complications are a major cause of morbidity and mortality in surgical patients. Each year, more than 10 million people worldwide suffer cardiac events (e.g., myocardial infarction or cardiac arrest) within 30 days of major noncardiac surgery [6]. The stress of surgery - especially in older patients with comorbid cardiovascular disease - can precipitate myocardial ischemia, heart failure, and arrhythmias. This review focuses on general and orthopedic surgeries, examining the epidemiology, risk factors, pathophysiology, risk stratification, diagnostic innovations, management, and guideline-based prevention of perioperative cardiac complications. Emphasis is placed on recent evidence (2015-2025), including biomarkers and improved risk indices, to inform guideline-concordant care.

Methods and search strategy

We conducted a narrative review following the Preferred Reporting Items for Systematic Reviews and Meta-Analyses (PRISMA) principles for transparent reporting. We searched PubMed/MEDLINE, Embase, Cochrane Library, and Scopus for studies published January 2015-October 2025, using combinations of the following terms: perioperative, non-cardiac surgery, cardiac complications, myocardial infarction, MINS, troponin, NT-proBNP, risk stratification, RCRI, AUB-HAS2, beta-blocker, statin, and guideline. Searches were last updated on October 14, 2025; records were limited to human adults and the English language. Reference lists of key articles were screened to identify additional studies, and landmark trials outside the date window (e.g., CARP 2004; POISE 2008) were purposefully included due to ongoing relevance.

The following are the inclusion criteria: peer-reviewed randomized trials, prospective/retrospective cohorts, systematic reviews/meta-analyses, and major society guidelines in adult non-cardiac surgery. The following are the exclusion criteria: case reports/series, editorials, non-peer-reviewed web sources, pediatric populations, or studies without perioperative cardiac outcomes/risk assessment.

Study Selection and Data Extraction

Two reviewers independently screened records and extracted data; disagreements were resolved by consensus.

Quality Considerations

We qualitatively considered risk of bias (randomization/confounding, blinding, outcome ascertainment) and the certainty of evidence; given heterogeneity, we performed a narrative synthesis rather than meta-analysis.

This review is narrative. A formal meta-analysis was not performed because of substantial heterogeneity across studies in design, patient selection, perioperative settings, biomarker assays and thresholds, and outcome definitions. Where available, we report standardized effect measures (RR, OR, or HR) with 95% confidence intervals from the original publications. This narrative review was not prospectively registered; risk of bias was qualitatively considered (randomization/confounding, blinding, outcome ascertainment).

Epidemiology

MACE - commonly defined as myocardial infarction, cardiac arrest, or cardiac death - occur in approximately 1-5% of patients undergoing major noncardiac surgery, varying with patient risk and surgical type [6,7]. In the United States, one large cohort study reported an overall perioperative myocardial infarction incidence of about 0.3%, but with much higher rates in high-risk surgeries (e.g., ~1-2% in vascular and thoracic procedures) [6]. In patients over 45 undergoing inpatient non-cardiac surgery, the incidence of MINS has been reported around 8% [6]. Notably, most of these perioperative infarctions are clinically silent - one analysis found that >80% of patients with a perioperative MI did not experience chest pain [8]. Nonetheless, MINS carries a significant prognostic impact, with an estimated 30-day mortality of 8-10% [6]. Orthopedic surgeries, particularly in frail elderly patients, also carry substantial cardiac risk. For example, urgent hip fracture repair in older adults is associated with perioperative cardiac complication rates around 5-10%, markedly higher than elective orthopedic procedures [9]. Overall, as surgical volumes increase with an aging population, perioperative cardiac events represent a significant public health concern [6].

Risk factors

Patient-specific factors play a central role in perioperative cardiac risk. Established cardiac history - such as prior coronary artery disease, heart failure, cerebrovascular disease, or high-risk arrhythmias - markedly increases the likelihood of perioperative MACE [8]. Other comorbidities, including diabetes, chronic kidney disease, and uncontrolled hypertension, further compound risk. Age is a major contributor, not only as a proxy for atherosclerosis but also due to decreased physiological reserve. Frailty, a syndrome of diminished functional reserve in older patients, has emerged as an independent predictor of worse postoperative outcomes. Even after adjusting for age and comorbidities, frail surgical patients have higher rates of cardiac complications, prolonged hospital stay, and mortality. In one systematic review, frailty was more predictive of 30-day and one-year postoperative mortality than chronological age [10].

Surgery-specific factors are equally important. The urgency and magnitude of the operation significantly influence risk. Emergency surgeries (e.g., for ruptured abdominal organs or hip fractures) carry a higher cardiac risk than elective procedures, due to limited time for optimization and the intense physiological stress of acute illness [9]. High-risk surgical categories include major vascular surgeries (e.g., aortic aneurysm repair), which have an estimated >5% risk of MACE, as well as lengthy intraperitoneal or intrathoracic operations. Orthopedic surgeries are generally intermediate-risk (~1-5% MACE), but within this category, an urgent hip fracture surgery in a frail elderly patient may approach the risk levels of vascular surgery [11]. By contrast, low-risk surgeries (e.g., superficial procedures, cataract surgery) have <1% risk of cardiac events [8].

Clinical risk indices have been developed to quantify patient/surgical risk factors into a score. The RCRI remains one of the most widely used tools. The RCRI assigns one point for each of six predictors: high-risk surgery, history of ischemic heart disease, history of heart failure, history of stroke or TIA, insulin-dependent diabetes, and chronic renal insufficiency (Cr >2 mg/dL). An RCRI score ≥2 indicates elevated risk of perioperative cardiac events. In contemporary practice, the RCRI has limitations in calibration, so newer models incorporate additional factors. The American College of Surgeons NSQIP risk calculator is an alternative, which was derived from a nationwide surgical database and includes variables such as age, functional status, and procedure-specific factors to predict risk of cardiac arrest or MI [12]. More recently, biomarker-enhanced indices have shown promise. For instance, the AUB-HAS2 cardiovascular risk index (developed at the American University of Beirut) combines clinical factors and laboratory markers. It assigns points for Age, Urgent surgery, history of Bypass (or CAD), Heart failure, ASA class, and prior Stroke (with stroke weighted 2 points) [13]. The AUB-HAS2 score stratifies patients into low, intermediate, and high risk groups and has demonstrated improved discrimination for predicting 30-day cardiac events compared to RCRI. Such integrated tools underscore the multifactorial nature of perioperative cardiac risk assessment [14].

Pathophysiology

The perioperative period triggers a cascade of physiological stress responses that can precipitate cardiac complications. Surgical trauma and anesthesia lead to a surge in sympathetic tone, causing tachycardia and blood pressure fluctuations that increase myocardial oxygen demand while potentially reducing coronary perfusion. This supply-demand mismatch can result in type II myocardial infarction (ischemia due to increased demand or decreased supply without an acute plaque rupture). Additionally, the neurohormonal stress responses - including catecholamine release, cortisol surge, and inflammatory cytokines - create a prothrombotic and proinflammatory milieu. This can destabilize atherosclerotic plaques, sometimes precipitating type I MI (acute coronary thrombosis from plaque rupture) in the perioperative setting [8]. Hypercoagulability and endothelial activation post-surgery also contribute to the risk of coronary thrombosis and postoperative venous thromboembolism.

Importantly, the majority of perioperative myocardial infarctions are clinically silent. Due to analgesia and residual anesthesia, patients often do not experience chest pain even during significant ischemic events. In one cohort, only ~14% of patients suffering a perioperative MI reported chest pain. Most infarctions are detected only by biomarker elevation or ECG changes, explaining why routine monitoring is crucial. Perioperative MIs are frequently non-ST-elevation in nature (demand ischemia); ST-elevation MI (from acute plaque rupture) is less common but tends to present with hemodynamic instability when it does occur [9]. Beyond myocardial ischemia, the perioperative inflammatory state and electrolyte shifts can provoke arrhythmias. Atrial fibrillation (AF) is observed in a subset of surgical patients, particularly after thoracic or major abdominal procedures, due to stress, catecholamines, and possibly pericardial irritation in cardiothoracic cases. Postoperative AF can exacerbate hemodynamic instability and increase stroke risk. Similarly, fluid overload or transfusion-related stress may precipitate acute heart failure in susceptible individuals, while uncontrolled hypertension or severe anemia can strain the myocardium. In summary, perioperative cardiac complications usually result from an interplay of supply-demand ischemia, hypercoagulability, and hemodynamic stress on a vulnerable cardiovascular system.

Risk stratification

Functional Capacity (METs, CPET)

Thorough preoperative risk stratification is essential for identifying patients at risk and guiding management. Current guidelines (ACC/AHA and ESC) recommend an approach that integrates clinical risk factors with an assessment of functional capacity [15]. Patients are first stratified by surgical risk: low-risk procedures (e.g., superficial or endoscopic surgeries) generally do not require further cardiac workup, whereas higher-risk surgeries (intraperitoneal, intrathoracic, vascular, orthopedic hip fracture, etc.) warrant closer evaluation. If a patient has poor functional capacity (<4 METs, equivalent to inability to climb a flight of stairs) and possesses significant clinical risk factors (e.g., multiple RCRI factors), guidelines suggest considering noninvasive cardiac testing if the results would change management [8,15]. For instance, an intermediate-risk surgery patient with poor exercise tolerance and angina symptoms might undergo stress imaging to detect severe coronary disease that could be treated prior to surgery. In contrast, patients with moderate or good functional capacity can proceed to surgery with optimized medical therapy in most cases.

Clinical Risk Scores (RCRI, ACS-NSQIP, AUB-HAS2)

Risk prediction tools inform this decision-making. The RCRI stratifies patients into classes where ≥2 factors indicate elevated risk. The American College of Surgeons - National Surgical Quality Improvement Program (ACS-NSQIP) risk calculator (also known as the MICA calculator for MI or Cardiac Arrest) provides a percentage risk based on patient and procedure characteristics [16]. It has been shown to reclassify some patients’ risk more accurately than RCRI, especially in those with advanced age or poor functional status not captured by RCRI [17]. A summary of commonly used perioperative cardiac risk scores and their clinical application is presented in Table 1 [13,16].

**Table 1:** Clinical risk scores at a glance—RCRI, ACS-NSQIP/MICA, and AUB-HAS2 RCRI: Revised Cardiac Risk Index; ACS-NSQIP: American College of Surgeons - National Surgical Quality Improvement Program; MICA: Myocardial Infarct or Cardiac Arrest; AUB-HAS2: American University of Beirut - Cardiovascular Risk Index

Score	Inputs (summary)	Predicted outcome	Quick thresholds/classes	Notes						
RCRI (Revised Cardiac Risk Index)	High-risk surgery; ischemic heart disease; heart failure; cerebrovascular disease; insulin-treated diabetes; creatinine >2.0 mg/dL	30-day major cardiac complications (MI, cardiac arrest)	0 = low; 1 = low-intermediate; ≥2 = elevated	Widely used; may under-estimate risk in contemporary cohorts; first-pass screen						
ACS-NSQIP / MICA	Procedure + patient factors (age, functional status/ASA class, emergency, etc.) from the NSQIP dataset	Perioperative risk of MI or cardiac arrest (%)	Continuous % risk output	Often reclassifies risk vs RCRI, especially in older/complex patients						
AUB-HAS2	Age >75; Urgent surgery; prior Bypass/CAD; Heart failure; ASA class ≥3; prior Stroke (2 points)	30-day MACE	0-1 = low; 2-3 = intermediate; ≥4 = high	Improved discrimination vs RCRI in several surgical subgroups						

Biomarkers

In recent years, circulating biomarkers have dramatically improved cardiac risk stratification. B-type natriuretic peptide (BNP) or N-terminal proBNP levels measured prior to surgery are among the strongest independent predictors of perioperative cardiac events [18-21]. An elevated NT-proBNP signifies underlying cardiac strain (from ventricular dysfunction or ischemia) and has been linked to a higher risk of postoperative heart failure, MI, and 30-day mortality. A large international cohort study (VISION) found that NT-proBNP thresholds could stratify patients into low, intermediate, and high risk for 30-day cardiovascular complications [21]. In that study, a preop NT-proBNP ≥300 pg/mL (or even lower cutoffs in some analyses) was associated with a several-fold increase in risk of cardiac death or nonfatal MI. Similarly, even minor preoperative elevations of high-sensitivity cardiac troponin (hs-cTn) indicate underlying myocardial injury or stable troponin leak and portend higher postoperative event rates. Incorporating biomarkers into risk models adds significant predictive power beyond clinical variables alone [19]. For example, the METS study (2018) evaluated various methods of risk assessment in 1,400 patients and found that NT-proBNP levels were superior to subjective METs or even formal exercise testing in predicting 30-day cardiac complications [20].

Given this evidence, some guidelines now recommend biomarker-based stratification for select patients. The 2017 Canadian Cardiovascular Society guidelines advise measuring NT-proBNP preoperatively in patients over 65 or those 45-64 with significant risk factors, to guide the intensity of perioperative monitoring and management. An elevated NT-proBNP or troponin can trigger closer postoperative surveillance (e.g., telemetry, troponin checks) and prophylactic measures. New risk indices such as AUB-HAS2 incorporate these biomarkers alongside clinical data to refine risk estimation [21]. In summary, modern risk stratification uses a combination of scoring tools and biomarkers to identify high-risk patients who may benefit from further cardiac evaluation or tailored perioperative management. A biomarker-guided perioperative risk and surveillance approach (NT-proBNP, high-sensitivity troponin, and MINS detection) is presented in Table 2 [15,21].

**Table 2:** Biomarker-guided risk stratification and postoperative surveillance (NT-proBNP, hs-cTn, MINS)

Element	Typical thresholds/timing	What it predicts	Suggested actions (when positive)											
Pre-op NT-proBNP	Low <100 pg/mL; Intermediate 100-299 pg/mL; High ≥300 pg/mL (pre-op)	30-day cardiac complications (MI/HF/death); need for monitored care	Address reversible issues; higher-level monitoring; plan post-op troponin (48-72 h) + telemetry if high risk											
Pre-op hs-troponin	Above assay-specific 99th percentile or rising pattern (pre-op)	Underlying myocardial injury leads to higher perioperative events	Optimize ischemia control; escalate monitoring; consider cardiology input											
Post-op hs-troponin (MINS)	Troponin >99th percentile with rise/fall within 48-72 h post-op; often asymptomatic	Strong predictor of 30-day mortality and complications	Daily troponin x 48-72 h in high-risk; telemetry; treat per ACS principles; continue statin/aspirin as indicated; HR/BP control											
Who to monitor (candidates)	Age≥65 or 45-64 with multiple risk factors/poor METs; elevated pre-op NT-proBNP; RCRI ≥​​​​​​​1-2; major surgery	Identifies those most likely to benefit from surveillance	Telemetry + hs-cTn 48-72 h; early cardiology if dynamic troponin or ECG changes											
Therapeutic implication (MINS)	Consider DOAC in selected MINS patients after hemostasis	Lower composite CV events (long-term)	Shared decision; ensure hemostasis; close follow-up											

Diagnostic innovations

Early detection of perioperative cardiac injury has improved with advances in monitoring and biomarkers. High-sensitivity troponin (hs-cTn) assays enable the detection of even small amounts of myocardial injury that were previously below the threshold of conventional troponin tests. Routine postoperative troponin surveillance has therefore emerged as a strategy to identify asymptomatic MIs (MINS). Studies have shown that applying daily troponin measurements in the first two to three days after surgery can detect myocardial injury in a considerable proportion of at-risk patients, allowing for prompt intervention [22]. For example, in the VISION cohort, patients with peak postoperative hs-cTnT above the 99th percentile had significantly higher 30-day mortality (approximately a 10-fold increase) despite most lacking ischemic symptoms. Recognizing this “silent” ischemia has spurred changes in practice. The 2017 Canadian guidelines recommend postoperative troponin monitoring for 48-72 hours in patients over age 65 or those with RCRI ≥1 undergoing major surgery. European guidelines have similarly noted that troponin surveillance may be considered in high-risk patients, as it can identify MINS and facilitate secondary prevention measures [23].

Improvements in perioperative monitoring technology have also enhanced diagnostic capabilities. Continuous telemetry is commonly utilized for patients with known cardiac disease or those who develop concerning signs postoperatively. Modern telemetry systems with automated ST-segment analysis can provide early warning of ischemia or arrhythmias even when clinicians are not present at the bedside. For high-risk surgeries (e.g., major vascular or thoracic procedures), some centers employ continuous 12-lead ST-segment monitoring in the ICU setting, which has been shown to increase detection of ischemic events compared to standard care [24]. Additionally, point-of-care cardiac ultrasound (focused transthoracic echocardiography) is increasingly available to anesthesiologists and intensivists. A quick bedside echo can help differentiate causes of hemodynamic instability - for instance, identifying new wall motion abnormalities (suggesting MI), cardiac tamponade, or acute heart failure - and guide appropriate management within minutes.

Another innovation is the use of biomarkers such as BNP in the postoperative period for risk stratification of complications. Postoperative elevations of BNP or NT-proBNP (measured on postoperative days one to three) have been associated with increased likelihood of heart failure, AF, and even 30-day mortality [3]. While not yet routine, some protocols include a postoperative BNP measure in patients who had elevated pre-op levels, to decide on the level of care (e.g., ward vs ICU) and aggressive diuresis or other measures. Diagnostic algorithms are also being refined - for instance, using delta changes in troponin to distinguish acute perioperative MI from chronic elevations. The Fourth Universal Definition of MI (2018) provides specific criteria for perioperative MI, requiring a rise and/or fall of troponin plus supporting evidence of ischemia (symptoms, ECG changes, imaging, or angiographic findings) to diagnose an acute MI. This helps avoid overdiagnosis in patients with stable troponin elevation. Overall, the combination of advanced biomarker monitoring and noninvasive imaging modalities is significantly improving our ability to promptly diagnose perioperative cardiac complications.

Management of perioperative cardiac complications

Management strategies span the preoperative, intraoperative, and postoperative phases to mitigate cardiac risk.

Preoperative Optimization and Medical Management

Preoperative optimization is aimed at stabilizing known cardiac conditions before surgery [25-27]. Guidelines emphasize that patients with unstable coronary artery disease (e.g., recent acute coronary syndrome) or decompensated heart failure should have surgery postponed if possible until those conditions are treated or stabilized. In practice, this means that a patient presenting for elective surgery who has unstable angina or acute HF would first receive medical therapy (or revascularization for acute coronary syndrome) and delay surgery for days to weeks. There is no benefit to prophylactic revascularization in stable patients without an ACS indication - the landmark CARP trial demonstrated that performing routine preoperative coronary stenting or bypass in stable patients undergoing vascular surgery did not improve outcomes compared to optimal medical therapy [28,29]. Thus, revascularization is reserved for those who meet standard cardiology indications independent of the surgery.

Medical optimization involves ensuring adequate beta-blockade, statin therapy, and blood pressure control in appropriate patients. If a patient is already on beta-blockers, these should be continued throughout the perioperative period (withdrawal can trigger rebound tachycardia and hypertension) [30-34]. Initiation of beta-blockers de novo is more nuanced. High-dose beta-blocker started immediately before surgery has been linked to adverse outcomes (as seen in the POISE-1 trial, where aggressive titration on the day of surgery increased stroke and hypotension). Current best practice is to start beta-blockers in high-risk patients well in advance (weeks) of surgery, at low doses, and up-titrate to achieve heart rate control, thereby reducing ischemic risk without causing hemodynamic instability. A 2018 Cochrane review found that perioperative beta-blockers did reduce nonfatal MI by ~27% but at the cost of a 73% increase in stroke and significant bradycardia/hypotension [26]. This underscores the importance of careful patient selection and dosing. Patients with multiple risk factors (e.g., RCRI ≥3 or positive stress test) might benefit from beta-blockers, whereas low-risk patients should not be routinely started on them.

Statins have cardiovascular protective effects (plaque stabilization, anti-inflammatory) and are recommended for most patients with elevated cardiac risk. Guidelines advise continuing chronic statin therapy and initiating a statin in statin-naïve patients who are undergoing high-risk procedures (particularly vascular surgery) or who have clinical indications (e.g., LDL above targets). Ideally, statins should be started at least a few weeks before surgery to confer benefit. Randomized data specific to the perioperative window are limited; one trial of high-dose atorvastatin started the day before vascular surgery (the LOAD trial) did not show a significant reduction in cardiac events. However, observational evidence suggests patients on long-term statins have fewer perioperative MIs, and statins likely improve overall outcomes in those with risk factors. Therefore, for a patient with, say, diabetes or prior CAD undergoing joint replacement, starting a moderate-dose statin well before surgery is a reasonable protective strategy. Other medications are managed strategically: ACE inhibitors/ARBs are often held on the day of surgery to avoid intraoperative hypotension (this practice is supported by some data showing reduced vasopressor requirement when ACEi/ARBs are skipped the morning of surgery) [3]. Antiplatelet and anticoagulant therapy management requires balancing bleeding versus thrombotic risk - as a rule, aspirin for secondary prevention is continued for most surgeries (exceptions being neurosurgical or other very high-bleeding-risk procedures), whereas dual antiplatelet therapy (DAPT) or anticoagulants are temporarily interrupted according to guidelines and bridging protocols if necessary.

Intraoperative Monitoring Strategies

Intraoperative management focuses on meticulous hemodynamic control and myocardial oxygen balance. Anesthetic technique should ensure adequate depth to blunt sympathetic surges during intubation and surgical stimulation but also avoid excessive vasodilation or myocardial depression. Continuous arterial pressure monitoring is used in high-risk patients to promptly treat hypotension or hypertension. Tachycardia is particularly deleterious (increasing oxygen demand and decreasing diastolic coronary perfusion); thus, beta-blockers (e.g., esmolol) or deeper anesthesia are used to keep heart rates in a moderate range. Conversely, significant hypotension is treated with vasopressors and fluids to maintain coronary perfusion pressure [27]. There is growing recognition that even brief periods of intraoperative hypotension (e.g., MAP <65 mmHg) are associated with myocardial injury and acute kidney injury; anesthesiologists now often follow “blood pressure targets” to minimize such episodes. In major surgeries, transesophageal echocardiography (TEE) can be employed intraoperatively to monitor cardiac function and volume status in real time - for example, detecting new wall motion abnormalities suggesting ischemia, or guiding volume resuscitation to avoid fluid overload. The use of regional anesthesia techniques (epidurals or nerve blocks) in conjunction with general anesthesia can attenuate the stress response to surgery and provide better pain control, which may indirectly reduce cardiac strain. Additionally, measures to reduce blood loss (minimally invasive surgical approaches, antifibrinolytics such as tranexamic acid, controlled hypotension techniques in select cases) can prevent anemia and the need for transfusion, thereby lowering the risk of demand ischemia.

Postoperative Monitoring and MINS Management (MANAGE)

Postoperative management is crucial for mitigating the impact of cardiac complications when they occur. Early detection is addressed above - continuous monitoring and troponin checks in high-risk patients. When a myocardial infarction is diagnosed postoperatively, treatment should follow acute coronary syndrome guidelines with modifications for the surgical context. Hemodynamically unstable ST-elevation MI (STEMI) is an indication for emergent reperfusion therapy; in the perioperative setting, this usually means urgent percutaneous coronary intervention (PCI) because thrombolysis is often contraindicated due to recent surgery [15]. Multidisciplinary consultation is vital - involving cardiology and the surgical team to manage antithrombotic therapy in the context of surgical bleeding risk. For non-ST elevation MI or MINS, a more conservative strategy is often employed initially: intensive medical therapy with oxygen, analgesia, beta-blockers [28] (if tachycardic or hypertensive), and aspirin and statin therapy is started or continued [6]. If troponin elevations are mild and the patient is stable, one might delay invasive assessment until the patient can better tolerate DAPT. However, if there is significant myocardium at risk or persistent ischemia, an early (within a few days) angiography and revascularization might be pursued, balancing bleeding risk (often after one week post-surgery, the bleeding risk diminishes enough to safely proceed with PCI and DAPT) [29,30].

One emerging therapy for MINS is anticoagulation to prevent secondary events. The MANAGE trial (2018) tested dabigatran 110 mg BID in patients who had MINS and found a reduction in major cardiac and cerebrovascular events over two years (a composite of cardiovascular death, MI, stroke, etc.), with an absolute risk reduction of about 4%. While bleeding was increased, it was not statistically significant in that trial [23,35]. This suggests that, in patients with substantial myocardial injury but who did not otherwise qualify for therapeutic anticoagulation, a direct oral anticoagulant (DOAC) may confer benefit. It is not yet standard of care, but some experts will consider anticoagulation (or intensified antithrombotic therapy such as adding low-dose rivaroxaban or aspirin) in a patient with a significant postoperative troponin elevation after weighing bleeding risk.

Arrhythmia and heart failure management

Management of arrhythmias in the postoperative period follows typical advanced cardiovascular life support (ACLS) protocols with adjustments. New-onset AF is common, particularly after cardiothoracic and major abdominal surgeries. Rate control with beta-blockers or non-dihydropyridine calcium blockers is the usual first step. If AF causes hemodynamic instability, synchronized cardioversion is indicated. Many postoperative AF episodes are transient and related to stress or electrolyte abnormalities; often, they convert to sinus rhythm within 24-48 hours. Anticoagulation for AF is a challenge - if AF persists beyond 48 hours or is highly symptomatic, and the bleeding risk permits, anticoagulation should be initiated to reduce stroke risk (especially if the patient’s CHA₂DS₂-VASc score is ≥2). Some patients may receive short-term anticoagulation and then reassessment for need if the AF resolves. Ventricular arrhythmias post-op are usually in the context of severe ischemia or electrolyte disturbances and are managed with antiarrhythmics and correcting the underlying cause, along with possible urgent revascularization if due to MI.

Acute heart failure or cardiogenic shock after surgery demands prompt supportive care. This includes diuresis for acute volume overload, afterload reduction (carefully, with IV vasodilators such as nitroglycerin if blood pressure tolerates), and inotropic support for cardiogenic shock (e.g., dobutamine or epinephrine infusions). Noninvasive or invasive ventilation may be required for pulmonary edema. In extreme cases of cardiogenic shock (e.g., from a massive MI), mechanical circulatory support (intra-aortic balloon pump or temporary ventricular assist device) is considered, though this is rare and would only be pursued in an ICU/cardiac critical care setting. Immediate consultation with cardiology is warranted for acute heart failure or shock. Co-management by cardiologists and intensivists can improve outcomes, as they can guide advanced therapies and the timing of any cardiac interventions.

Finally, secondary prevention is addressed once the patient recovers from the acute issue. A perioperative cardiac complication serves as a prognostic warning - patients have an elevated long-term risk of mortality and cardiovascular events [22]. Thus, at discharge or soon after, they should be optimized on chronic cardioprotective medications (aspirin, statin, beta-blocker, ACE inhibitor as appropriate) and referred for cardiac rehabilitation if feasible. Ensuring tight control of diabetes, hypertension, and other risk factors is also critical. In essence, the management of perioperative cardiac complications extends beyond the hospital stay, transitioning into standard chronic cardiovascular care to improve long-term outcomes. Quantitative findings from major trials and cohorts are summarized in Table 3 using standardized effect measures (RR/OR/HR with 95% CIs) to enhance transparency.

**Table 3:** Key effects summary from major trials and cohorts (effect measure and 95% CI) MINS: Myocardial Injury After Non-cardiac Surgery; MACE: Major Adverse Cardiac Events; MI: Myocardial Infarction

Study (Year)	Population/Intervention or Exposure	Primary Endpoint	Effect Measure (95% CI)	Principal Conclusion										
VISION (2012) [21]	>=45 y adults undergoing inpatient noncardiac surgery; postoperative hs-TnT measured	30-day all-cause mortality	Approx. mortality ~10% with MINS vs ~1% without; adjusted association reported in source (exact OR/HR per troponin strata in JAMA 2012)	Postoperative troponin elevation (MINS) strongly associated with increased 30-day mortality										
METS (2018) [32]	1,401 elevated-risk adults; compared functional capacity measures and NT-proBNP	30-day cardiac complications	NT-proBNP outperformed subjective METs/CPET; incremental discrimination reported in source (c-statistics)	Pre-op NT-proBNP is the strongest single predictor of 30-day cardiac events										
JAMA (2017) hs-troponin [31]	Adults undergoing noncardiac surgery; postoperative hs-Tn categories	30-day mortality / MINS	Risk increases across hs-Tn categories; exact adjusted HR/OR by category in source	Even modest postoperative hs-Tn elevations predict higher 30-day mortality										
Duceppe (2020) [36]	10,402 adults; pre-op NT-proBNP thresholds	30-day CV complications	High NT-proBNP (>=300 pg/mL) associated with higher event rates; exact adjusted effect sizes in source	Pre-op NT-proBNP stratifies risk; low values identify very low-risk patients										
Zhao (2024) [37]	Major noncardiac surgery; pre-op NT-proBNP + hs-cTnT	Composite MACE	Both biomarkers independently predicted outcomes; exact adjusted HRs in source	Combining NT-proBNP with hs-cTn improves pre-op risk prediction										
Martinez-Perez (2024) [38]	High-risk surgical patients; routine post-op troponin surveillance	Detected perioperative MI / risk stratification	Surveillance identified ~1/5 with silent injury; modeling results in source	Routine surveillance detects otherwise silent MI and refines early risk										
POISE (2008) [39]	Extended-release metoprolol vs placebo perioperatively	Composite CV outcomes; MI; stroke	Reduced MI but increased stroke with metoprolol (exact RRs in Lancet 2008)	Beta-blockade reduces MI but increases stroke when initiated late/high dose										
Cochrane beta-blockers (2019) [26]	83 trials; perioperative beta-blockers	MI; AF; stroke; mortality	MI decrease ~28% (RR~0.72); AF decrease ~60%; stroke increase ~65% (RR~1.65); no mortality benefit (see Cochrane for exact CIs)	Benefits in ischemia/AF offset by increased stroke; careful selection/titration required										
LOAD (2017) [34]	High-dose atorvastatin loading vs placebo in vascular surgery	Cardiac events	No significant difference vs placebo	Perioperative statin loading did not reduce events; continue/indicated initiation still recommended										
MANAGE (2018) [35]	Dabigatran 110 mg BID vs placebo in MINS	Composite CV events	HR ~0.72 (95% CI ~0.55-0.93) for primary composite	Dabigatran reduced composite CV events in selected MINS patients without significant increase in major bleeding										

Guideline-based recommendations (AHA/ACC 2024; ESC 2022)

Evidence-based guidelines from professional societies synthesize the above principles into structured algorithms for perioperative cardiac care. The 2024 AHA/ACC Multisociety Guideline and the 2022 ESC guideline are key references for current practice [5,15]. Both guidelines advocate a stepwise approach:

Step 1: Determine urgency of surgery. If the surgery is an emergency, there is no time for formal cardiac testing; proceed to surgery with medical optimization and monitoring as able. If not an emergency, move to Step 2.

Step 2: Consider active cardiac conditions. Postpone elective surgery in the presence of unstable coronary syndromes, acute heart failure, severe arrhythmias, or valvular disease causing symptoms until those are treated. Urgent surgeries may proceed with appropriate intraoperative and postoperative precautions (e.g., invasive monitoring, postoperative ICU) while managing the condition.

Step 3: Estimate surgical risk. As discussed, categorize the procedure as low risk (<1% MACE risk) or elevated risk (>1%). Low-risk procedures (e.g., breast surgery, cataract, minor ambulatory surgery) generally require no further cardiac evaluation - they can go ahead in virtually all patients after routine assessment [27]. For elevated-risk surgeries (most major intra-abdominal, thoracic, vascular, orthopedic), assess the patient’s clinical risk factors.

Step 4: Assess the patient’s functional capacity and risk factors. If the patient has moderate or good functional capacity (≥4 METs, e.g., can climb stairs or perform heavy housework) and has no more than one or two minor risk factors, guidelines say it is reasonable to proceed to surgery without additional cardiac testing. If functional capacity is poor or indeterminate, and the patient has multiple risk factors (e.g., RCRI ≥2) for a high-risk surgery, consider noninvasive stress testing (e.g., an exercise or pharmacologic stress imaging) if the results would change management (for instance, if a positive test would lead to coronary revascularization or intensified therapy). Importantly, testing should not be done routinely - only if it will impact decision-making. The ESC 2022 guideline also highlights the role of NT-proBNP in risk assessment: an elevated preoperative NP may indicate the need for postoperative monitoring even if formal stress testing is not done.

Step 5: Implement guideline-directed medical therapy and risk reduction. All patients, regardless of testing, should have optimization of blood pressure (target <130/80 in hypertensives, avoiding surgery if SBP >180 uncontrolled), blood glucose (avoid hyperglycemia >180 mg/dL perioperatively), and anemia if present (consider iron/transfusion for Hb <8-10 depending on situation). Beta-blockers: continue them if the patient is already on them. If not on them, only start in those who meet criteria (e.g., ≥3 RCRI factors or inducible ischemia on testing), and start weeks in advance if possible [28]. Statins: indicated for patients with ASCVD or LDL above guideline thresholds, and specifically recommended by ACC/AHA for patients undergoing vascular surgery or those with ≥1 risk factor undergoing elevated-risk surgery. Ideally, initiate statin therapy at least two weeks pre-op (earlier if possible). ACE inhibitors (ACEi): if the patient has heart failure or uncontrolled hypertension, they should be on an ACEi per usual guidelines, but on the day of surgery, many centers hold ACEi/ARB to reduce hypotension risk [3]. Smoking cessation at least four to eight weeks prior to surgery is advised to reduce cardiopulmonary complications.

Step 6: Decide on further management or proceed. If noninvasive testing is done and shows extensive ischemia or high-risk findings, the care team should consider consulting cardiology for possible coronary angiography. The ACC/AHA guideline, however, clearly states that prophylactic revascularization is not indicated unless the patient would merit it by standard cardiology criteria (e.g., left main disease, triple vessel disease with low EF, etc.). Evidence from the CARP trial demonstrated no mortality benefit from routine preoperative coronary revascularization before major vascular surgery, supporting current guideline recommendations to reserve such interventions for patients with standard indications [25,29]. In practice, this means only a small subset of surgical patients end up getting preoperative PCI or CABG - and only if they likely needed it anyway. Most patients, even with positive stress tests, are managed medically through the surgical period. After any necessary interventions or optimizations, the patient proceeds to surgery with appropriate intraoperative monitoring and postoperative plans [30].

Step 7: Postoperative monitoring and management. Guidelines emphasize postoperative vigilance for high-risk patients. The ESC 2022 and Canadian 2017 guidelines both suggest measuring troponin in the first 48-72 hours after surgery for patients with elevated risk (e.g., those with NT-proBNP above the cutoff, or RCRI ≥2 undergoing major surgery). Detection of asymptomatic MINS should prompt cardiology follow-up and management (as per the MANAGE trial, consideration of anticoagulation or intensification of therapy). Early mobilization and appropriate thromboprophylaxis (to prevent venous thromboembolism) are also part of standard care that indirectly benefits cardiac outcomes by reducing immobilization-related complications.

Throughout this process, multidisciplinary collaboration is key. Involvement of anesthesiologists, cardiologists, and surgeons in perioperative planning (often via a formal perioperative medicine service or “heart team” approach) has been shown to improve risk assessment and adherence to best practices. For instance, if a high-risk patient is identified, the team might opt for postoperative ICU monitoring or adjust the surgical plan (maybe staging procedures, or choosing regional anesthesia if appropriate). Studies have found that when guideline-based protocols are implemented - such as checklists for continuing beta-blockers and statins, or algorithms for hemodynamic management - the incidence of cardiac complications can be reduced [27]. Guideline-based care thus ensures that known effective measures (e.g., beta-blocker continuation and judicious use of testing) are consistently applied, while avoiding interventions that are unproven or harmful (e.g., inappropriate prophylactic surgeries or last-minute high-dose therapies). In summary, adherence to ACC/AHA and ESC guidelines provides a structured, evidence-backed framework to maximize patient safety with regard to cardiac events in the perioperative period.

Interpretation and areas of uncertainty: While biomarkers (NT-proBNP, hs-troponin) consistently stratify risk across settings, the optimal scope of routine postoperative troponin surveillance remains debated (resource use vs. detection of clinically silent injury). Perioperative β-blockade reduces myocardial ischemia in selected high-risk patients but can increase stroke and hypotension if initiated late or dosed aggressively; thus, continuation (not initiation on the day of surgery) and early, low-dose titration in appropriate candidates are favoured. Statins are generally continued and often initiated in indicated patients ahead of high-risk surgery, although perioperative loading has not shown consistent benefit. For MINS, anticoagulation (e.g., MANAGE) suggests benefit in carefully selected patients once hemostasis is secure. Overall, individualized risk assessment and context-specific application of guidelines remain essential.

**Figure 1:**
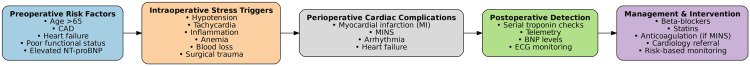
Conceptual framework illustrating the identification, prevention, monitoring, and intervention pathways in the management of perioperative cardiac complications The diagram emphasizes the integration of risk stratification tools, biomarker-based surveillance, hemodynamic monitoring, and timely therapeutic interventions across the surgical timeline to reduce morbidity and mortality in patients undergoing major noncardiac surgery. The diagram emphasizes the integration of risk stratification tools, biomarker-based surveillance, hemodynamic monitoring, and timely therapeutic interventions across the surgical timeline to reduce morbidity and mortality in patients undergoing major noncardiac surgery.

Figure 1 offers a conceptual framework of integrated assessment and management in the perioperative period.

## Conclusions

A practical approach is to use a stepwise algorithm anchored in clinical risk and functional capacity, adding NT-proBNP and high-sensitivity troponin selectively to refine risk in appropriate patients. Other approaches are to continue chronic beta-blockers; avoid day-of-surgery initiation at high doses; and consider early, low-dose titration only in carefully selected high-risk patients. Statins should be continued and initiated when indicated, recognizing that last-minute loading has not consistently shown benefit. For postoperative troponin elevation (MINS), ensure surveillance, optimize secondary prevention, and consider anticoagulation in carefully chosen patients once bleeding risk is acceptable.

Across the perioperative course, prioritize hemodynamic stability - avoiding prolonged hypotension and tachycardia - and tailor the intensity of monitoring to the individual’s risk profile. Guideline recommendations (AHA/ACC 2024; ESC 2022) should be applied with patient-specific nuance rather than as universal protocols, emphasizing individualized assessment to maximize safety and outcomes.
